# Differences in Opioid Prescribing by Urban and Rural Pharmacists in Nova Scotia, Canada—A Time Series Analysis from 2018 to 2022

**DOI:** 10.3390/pharmacy14030066

**Published:** 2026-04-29

**Authors:** Edward Chisholm, Ying Zhang, Chiranjeev Sanyal

**Affiliations:** 1 Department of Mathematics and Statistics, Acadia University, Wolfville, NS B4P 2R6, Canada; 2 College of Pharmacy, Dalhousie University, Halifax, NS B3H 4R2, Canada

**Keywords:** pharmacists, scope of practice, opioids, analgesic, rural, urban

## Abstract

During the COVID-19 pandemic, Health Canada temporarily exempted pharmacists from specific restrictions under the Controlled Drugs and Substances Act (CDSA), allowing them to prescribe opioids. However, it is not yet established whether opioid dispensing patterns differ between urban and rural pharmacists. This study aims to assess the impact of the CDSA subsection 56(1) temporary exemption on opioid prescribing practices among urban and rural pharmacists between 1 February 2018 and 30 April 2022. Descriptive statistics and visualizations assessed differences in opioid prescribing between urban and rural pharmacists under the CDSA exemption. Initial analyses employed linear regression to examine changes, followed by evaluation of temporal dependence using autocorrelation and residual analysis. When appropriate, a suitable time series model was subsequently applied. Following the CDSA exemption, the mean weekly proportion of opioid claims prescribed by urban pharmacists increased from 0.0% to 1.03%. In contrast, rural pharmacists’ prescriptions rose from 0.0% to 0.35%. The estimated mean level change was 0.667% for urban pharmacists (95% CI: 0.520–0.838%, *p* < 0.0001) and 0.201% for rural pharmacists (95% CI: 0.140–0.291%, *p* < 0.0001). The study identified distinct differences in opioid prescribing practices between urban and rural pharmacists in Nova Scotia. Furthermore, opioid prescriptions increased steadily across all patient groups, indicating evolving patterns of opioid use within the province.

## 1. Introduction

The opioid crisis in Canada constitutes a complex and evolving public health emergency, characterized by changing patterns of drug use, heightened toxicity in the illicit drug supply, and ongoing strain on healthcare systems [1]. From January 2016 to March 2022, 30,843 apparent opioid toxicity deaths and 32,319 opioid-related poisoning hospitalizations were recorded nationwide, highlighting the substantial burden of opioid-related harm [2]. Provincially, Nova Scotia has also been significantly affected, with 861 opioid-related deaths reported between 2009 and 2023 [3]. Although historically lower than rates in some western provinces, recent data indicate increasing vulnerability within Nova Scotia.

The COVID-19 pandemic marked a significant turning point in the progression of the opioid crisis. From April 2020 to March 2022, opioid toxicity deaths in Canada increased by 91%, accompanied by a 24% rise in opioid-related hospitalizations compared to the preceding two years [2]. This escalation is attributed to several intersecting factors, including the increased prevalence of highly potent synthetic opioids such as fentanyl in the drug supply and disruptions to healthcare and harm reduction services resulting from public health restrictions. These changes reduced access to care and disproportionately impacted individuals who use drugs, thereby elevating the risk of overdose and adverse outcomes.

Historically, prescription opioids have contributed significantly to opioid-related harms in Canada, primarily due to patterns of overprescribing and subsequent dependence [4]. Recent evidence demonstrates a shift in the primary drivers of overdose deaths, with illicit synthetic opioids such as fentanyl now accounting for most fatalities. This transition underscores changes in the drug supply and emphasizes the necessity for public health interventions that address both prescribing practices and the increasing risks associated with unregulated substances.

Within this evolving context, pharmacists have assumed a more significant role in maintaining access to care. In Nova Scotia, pharmacist-issued opioid prescriptions increased from 2213 between April 2020 and March 2021 to 3476 between April 2021 and March 2022, representing a 57% rise [5]. This increase likely reflects both the expanded scope of practice and greater reliance on pharmacy services during the pandemic, when access to other healthcare providers was limited. Although this shift may have supported continuity of care, particularly for pain management and opioid agonist therapy, it highlights the necessity for ongoing monitoring to ensure safe and appropriate use.

On 19 March 2020, in response to disruptions caused by the COVID-19 pandemic, Health Canada issued a nationwide class exemption under subsection 56(1) of the Controlled Drugs and Substances Act [6]. This policy sought to maintain continuity of care for individuals dependent on controlled substances, including opioids, during a period of severely restricted access to in-person healthcare. Public health restrictions, clinic closures, and reduced prescriber availability created significant barriers to care, thereby increasing the risk of treatment interruptions, withdrawal symptoms, unmanaged pain, and greater reliance on the illicit drug supply.

The exemption expanded pharmacists’ authority to manage controlled substances by permitting prescription extensions and renewals, transfers between pharmacies, and acceptance of verbal orders from prescribers [6,7]. Additionally, it enabled medication delivery to patients’ homes, thereby supporting adherence to public health measures. Implementation varied across provinces according to regulatory frameworks, resulting in differences in the adoption of these authorities [6,8]. This policy shift facilitated a more prominent role for pharmacists in patient care and medication management and raised important considerations for the long-term integration of expanded pharmacy services within the healthcare system.

The effectiveness of pharmacy-based services is influenced by both the scope of pharmacists’ practice and clinical expertise, as well as patients’ timely access to these services. Geographic accessibility serves as a critical determinant of healthcare utilization, especially for community pharmacy services that require in-person delivery. Studies from Ontario and Nova Scotia reveal significant disparities in pharmacy access between urban and rural populations, underscoring a key structural factor affecting care delivery [9,10].

Urban residents in both provinces have significantly greater access to pharmacies than those in rural areas. In Ontario, more than 70% of urban residents live within 800 m of a pharmacy, whereas fewer than 10% of rural residents have similar access, with comparable disparities at greater distances [9]. Nova Scotia demonstrates a similar trend, with over 60% of urban residents within 800 m of a pharmacy, compared to only 14% of rural residents [10]. These disparities highlight persistent challenges in healthcare access for rural communities and may affect the utilization of pharmacy services, including expanded prescribing roles, across different geographic settings.

Analyses of opioid prescribing patterns in the United States in 2021 indicate modest yet consistent differences between urban and rural populations. At the prescription level, metrics such as average days’ supply and the number of prescriptions per patient were broadly similar across both settings, suggesting comparable prescribing practices on an individual basis [11]. In contrast, total prescribing volumes were substantially higher in urban areas, a trend that primarily reflects differences in population size rather than prescribing behavior.

Population-adjusted analyses reveal a more nuanced pattern, with rural patients experiencing relatively higher opioid exposure. Rural individuals were slightly more likely to receive opioid prescriptions and had a greater mean total days’ supply compared to urban patients [12]. Additionally, rural patients were more likely to receive longer initial prescriptions, potentially increasing the risk of prolonged use and associated harms. These findings underscore the necessity of considering both absolute and population-adjusted measures when interpreting geographic differences in opioid prescribing.

During the COVID-19 pandemic, Health Canada implemented a temporary class exemption under subsection 56(1) of the Controlled Drugs and Substances Act to ensure continued access to controlled substances, including opioids, in response to widespread disruptions in healthcare services [6]. This policy broadened pharmacists’ authority to extend, renew, and manage prescriptions, with the objective of mitigating risks associated with treatment interruptions, such as unmanaged pain, withdrawal, and increased reliance on the illicit drug supply.

Despite the policy’s significance, empirical evidence regarding its effects on opioid prescribing across diverse geographic settings in Canada remains limited. Specifically, it is unclear whether the policy’s impact varied between urban and rural communities, where disparities in healthcare access and patient needs are well established. While previous time series analyses have documented overall increases in opioid prescribing during the pandemic [13], these studies have not assessed variation by pharmacist location, resulting in a gap in understanding geographic differences in policy uptake and impact.

This study evaluated the consequences of subsection 56(1) of the Controlled Drugs and Substances Act’s temporary exemption on opioid prescribing by pharmacists in urban and rural settings during the COVID-19 period. Prescribing trends were analyzed over a four-year period from 1 February 2018 to 30 April 2022, covering both pre-pandemic and pandemic phases, to determine how expanded pharmacist scope of practice influenced prescribing patterns and access to care across different geographic contexts.

## 2. Materials and Methods

The study was conducted in accordance with the Declaration of Helsinki, and approved by the Institutional Review Board of Dalhousie Health Sciences Research Ethics Board (protocol code: #2024-7130, approval date: 12 April 2024). Patient consent was waived due to this study’s use of secondary data analysis and does not require informed consent.

### 2.1. Study Population

In Nova Scotia, approximately 1598 licensed pharmacists deliver care in community and other healthcare settings, fulfilling essential roles in medication management, including dispensing, clinical assessment, and prescribing within an expanded scope of practice [14]. Data for this study were sourced from the Nova Scotia Prescription Monitoring Program, which records detailed information on controlled substances dispensed in community settings throughout the province [15,16]. Although this database serves as a valuable resource for monitoring prescribing trends, it excludes medications administered to hospital inpatients and thus primarily represents outpatient and community-based care.

The study population comprised adults aged 18 years and older in Nova Scotia who received opioid prescriptions from pharmacists. Geographic classification utilized Canada Post forward sortation areas, designating postal codes beginning with “B0” as rural and those beginning with “B1” to “B9” as urban [5]. This method serves as a practical proxy for geographic context, facilitating comparisons of prescribing patterns among populations with varying levels of healthcare access and service availability.

### 2.2. Data Source

The Nova Scotia Prescription Monitoring Program (NSPMP) is a provincially mandated system that monitors controlled substances regulated under the Controlled Drugs and Substances Act, including opioids and other medications with potential for misuse [17,18]. The primary objective of the NSPMP is to promote safe and appropriate medication use while reducing the risk of misuse, diversion, and opioid-related harms [15]. The program facilitates surveillance, informs clinical decision-making, and guides policy development, thereby enhancing medication safety and public health outcomes in Nova Scotia.

The NSPMP collects comprehensive, real-time data on all monitored drugs dispensed through community pharmacies across the province, serving as a robust resource for evaluating prescribing patterns [18]. However, the program excludes medications administered to hospital inpatients and therefore represents only outpatient and community-based care. Participation is mandatory for both prescribers and pharmacists [19], which ensures high data coverage and consistency. Prescriptions are documented using standardized forms or electronic systems that are integrated into a centralized Drug Information System, facilitating accurate tracking and oversight of controlled substance use.

The NSPMP database provides comprehensive patient-level data on dispensed controlled substances, facilitating the analysis of prescribing and utilization patterns. The database includes variables such as patient demographics (e.g., age and gender), medication details (e.g., drug identification number and classification), and prescription-specific information (e.g., days’ supply, dispensing date, drug strength, dosage form, route of administration, and prescriber identifier). This dataset enables a thorough evaluation of opioid prescribing practices, encompassing dosage, duration, and formulation.

All data were de-identified prior to analysis to maintain patient confidentiality and adhere to ethical standards. Data from 1 February 2018 to 30 April 2022 were analyzed, encompassing both pre-pandemic and pandemic periods. This approach enabled the assessment of temporal trends and the potential effects of COVID-19–related policy changes on opioid prescribing.

### 2.3. Statistical Analysis

Weekly opioid prescription claim counts attributed to urban and rural pharmacists in Nova Scotia were standardized as proportions of total opioid prescribing activity. For each week, the number of claims prescribed by urban or rural pharmacists was divided by the total number of opioid claims dispensed across all prescribers in the province. This method enabled comparison of relative prescribing contributions over time while accounting for fluctuations in overall prescribing volume. Descriptive statistics, including means and standard deviations, summarized prescribing patterns. Time-series plots were generated to visualize trends and to identify potential shifts associated with key events.

A multi-stage modeling approach was implemented to evaluate temporal changes. Initially, linear regression models estimated changes in prescribing levels and trends over time, including potential shifts during the study period. These analyses offered a preliminary assessment of temporal patterns, which were subsequently examined to clarify changes in opioid prescribing across urban and rural settings.

The residuals from the initial regression model were subsequently analyzed using an integrated autoregressive moving-average (ARIMA) model, which is commonly employed for correlated time series. The model parameters, including autoregressive (AR), differencing (I), and moving-average (MA) components, were determined using established model identification, estimation, and diagnostic procedures.

A logarithmic transformation was applied to the percentage outcome variable prior to analysis to satisfy assumptions of normality and homoscedasticity. This transformation stabilized variance and enhanced model fit. Model selection was guided by the Akaike Information Criterion (AIC), with lower values indicating superior fit, and was further supported by diagnostic checks of residuals for independence, constant variance, and approximate normality.

The combined regression and ARIMA modeling approach enabled a robust evaluation of statistically significant changes in opioid prescribing patterns associated with the Controlled Drugs and Substances Act-related policy intervention, while appropriately accounting for underlying temporal dependence and autocorrelation in the data.

In the final time series regression framework, the estimated intercept and its associated standard error represent the mean level of the outcome variable, specifically the percentage of opioid prescription claims, across the entire study period. These parameter estimates were subsequently used to calculate confidence intervals, which quantify the statistical uncertainty surrounding the overall mean proportion of claims attributed to urban and rural pharmacists in Nova Scotia.

The estimated regression coefficient for time, representing the slope parameter, and its standard error quantify the direction and magnitude of change in the percentage of opioid prescription claims over time. Confidence intervals constructed around this coefficient assess the precision and statistical significance of temporal trends, indicating whether observed increases or decreases in prescribing contributions are unlikely to have occurred by chance. This methodology is consistent with standard inferential techniques in time series analysis, where parameter estimates and their variability are used to evaluate both baseline levels and longitudinal changes in the outcome of interest.

Incorporating interval estimates enhances the robustness of interpreting prescribing patterns, facilitating comparisons between urban and rural pharmacists while accounting for sampling variability and model uncertainty. Collectively, these measures enable a comprehensive assessment of both the overall level and the rate of change in opioid prescribing following policy modifications under the Controlled Drugs and Substances Act.

A comprehensive set of diagnostic procedures was implemented to assess the adequacy of the final time-series models. Residual normality was assessed using normal probability plots and the Shapiro–Wilk test. Temporal independence was evaluated through autocorrelation and partial autocorrelation plots, as well as the Ljung–Box test. Homoscedasticity was examined using residual-versus-fitted plots. The results indicated that the key model assumptions of normality, independence, and constant variance were adequately satisfied.

Separate models were specified for urban and rural prescribing patterns, with model selection guided by standard diagnostic procedures and model fit statistics. ARIMA models were used to account for temporal dependence in the data. All statistical tests were two-tailed, with *p*-values < 0.05 considered statistically significant, and analyses were conducted using R (version 4.3.2).

## 3. Results

### 3.1. Mean Weekly Percentage of Claims

Following the implementation of the subsection 56(1) exemption under the Controlled Drugs and Substances Act, the proportion of opioid prescriptions dispensed by pharmacists in Nova Scotia increased in both urban and rural areas. In the pre-policy period (1 February 2018 to 18 March 2020), the mean weekly proportion was 0.0% for both groups, reflecting restricted prescribing authority. In the post-policy period (19 March 2020 to 30 April 2022), this proportion rose to 1.03% (SD 0.4%) for urban pharmacists and 0.35% (SD 0.2%) for rural pharmacists. These findings demonstrate uptake of expanded prescribing roles, with a greater increase observed in urban settings.

### 3.2. Scatter Plots

**Figure 1:**
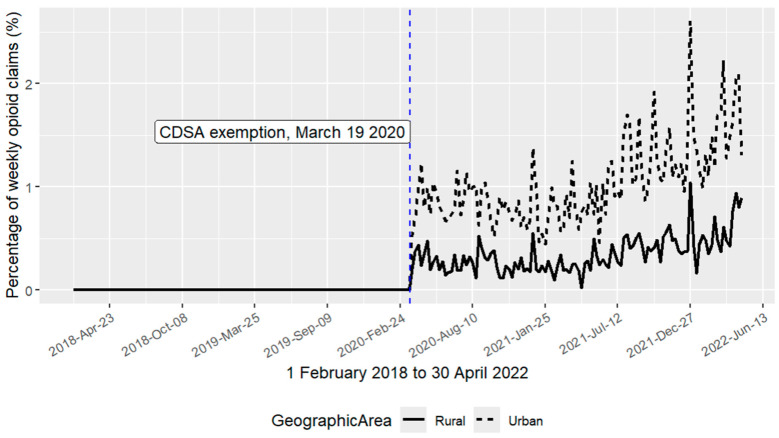
Percentage of weekly opioid prescriptions by urban and rural claims.

Figure 1 presents a scatter plot of weekly percentages of opioid prescription claims attributed to urban and rural pharmacists in Nova Scotia, illustrating distinct geographic differences in prescribing patterns following the subsection 56(1) Controlled Drugs and Substances Act exemption. During the post-policy period, urban pharmacists accounted for a higher proportion of claims than rural pharmacists, indicating greater adoption of expanded prescribing authority in urban areas. Both groups exhibit a clear upward trend beginning around June 2021.

### 3.3. Time Series Regression Models

Table 1 presents the results from the final time series regression models examining urban and rural prescribing patterns. For the urban dataset, the optimal model, as determined by the Akaike Information Criterion, was an ARIMA (5,0,0) model to address autocorrelation. Following the adjustment, a statistically significant increase in the mean weekly percentage of opioid prescription claims attributed to urban pharmacists was identified after the Controlled Drugs and Substances Act policy intervention. The estimated mean-level change was 0.667% (95% CI: 0.520–0.838%; *p* < 0.0001), representing an increase of approximately 0.5% to 0.8% of total claims.

In the context of rural prescribing, the optimal model incorporated an ARIMA (3,0,1) component, accounting for temporal dependence via autoregressive and moving-average terms. After adjustment, a statistically significant increase was identified, with an estimated mean-level change of 0.201% (95% CI: 0.140–0.291%; *p* < 0.0001). This change corresponds to an increase of approximately 0.1% to 0.3% of total opioid prescription claims during the post-policy period.

An analysis of slope parameters in urban and rural time-series models for Nova Scotia identified a statistically significant upward trend in the proportion of opioid prescription claims attributed to pharmacists over time. The estimated slope coefficients were 1.007 for urban and 1.009 for rural settings, corresponding to average weekly increases of approximately 0.01% in the proportion of total claims. These trends were statistically significant (urban: *p* = 0.0001; rural: *p* = 0.001), with 95% confidence intervals ranging from 1.00 to 1.01. This suggests that the observed increases are unlikely to be due to random variation.

## 4. Discussion

This study aimed to compare opioid prescribing by pharmacists in urban and rural populations in Nova Scotia, with a focus on their respective contributions to total opioid prescriptions. The analysis also examined how the expanded scope of practice and pandemic-related policy changes affected access to opioid medications in various geographic settings.

The findings demonstrate that prescribing patterns varied between urban and rural pharmacists. Urban pharmacists accounted for a higher proportion of opioid claims and exhibited a greater increase in claims following the Controlled Drugs and Substances Act subsection 56(1) exemption. These results indicate that geographic context influences the adoption of expanded prescribing authority. In contrast to previous U.S. research, which reported similar prescribing practices across settings, these findings may reflect differences in healthcare systems, pharmacist responsibilities, and access to care, especially during the COVID-19 pandemic.

In Nova Scotia, notable differences exist in opioid prescribing practices among pharmacists across geographic settings, with urban pharmacists responsible for a larger share of prescriptions compared to those in rural areas. This trend is likely influenced by higher population density, greater pharmacy availability, and increased patient demand in urban regions, which result in more frequent patient interactions and greater opportunities to exercise expanded prescribing authority under the Controlled Drugs and Substances Act subsection 56(1) exemption.

Rural pharmacists account for a smaller proportion of opioid prescriptions, likely due to factors such as smaller populations, limited pharmacy access, and distinct care delivery models. These results underscore the need to monitor the implementation of expanded prescribing authority in rural areas and to identify barriers to its adoption. Providing targeted support and resources could enhance pharmacists’ roles and promote equitable access to care across regions.

Addressing urban–rural disparities in pharmacist prescribing is essential to ensuring equitable access to healthcare services. Enhanced support for rural pharmacists can improve their capacity to utilize an expanded scope of practice, especially in areas where physician shortages lead to care gaps, longer wait times, and greater reliance on a small number of providers. Within this framework, pharmacists in rural Nova Scotia are positioned to play a critical role in sustaining access to opioid-related care following the Controlled Drugs and Substances Act subsection 56(1) exemption.

The lower level of prescribing observed among rural pharmacists may be attributed to differences in practice context and care delivery. Concerns about overuse, misuse, or unintended consequences associated with prescribing controlled substances may prompt rural pharmacists to adopt more conservative approaches, especially in smaller communities where professional accountability and patient familiarity are heightened [20,21]. Furthermore, limited access to interdisciplinary support, fewer clinical resources, and increased professional isolation may reinforce risk-averse decision-making. Therefore, these findings should be interpreted with caution, and additional research is required to elucidate the underlying factors contributing to geographic variation in pharmacist prescribing.

The findings underscore the necessity for targeted initiatives to support rural pharmacists in the safe and effective utilization of an expanded scope of practice. Future research should prioritize identifying barriers to the adoption of Controlled Drugs and Substances Act-related exemptions in rural contexts and evaluating interventions to address these challenges, including enhanced clinical training, improved access to mentorship and peer-support networks, and the creation of decision-support tools specifically designed for rural practice. Strengthening these supports may increase confidence and consistency in prescribing, thereby improving access to care and reducing geographic disparities.

In Nova Scotia and other Canadian jurisdictions, expanded prescribing privileges, enabled by policies such as the Controlled Drugs and Substances Act subsection 56(1) exemption and credentialing pathways like Advanced Practice Authority, have created new opportunities for pharmacists to assume broader clinical responsibilities. Although some evidence indicates that additional training and experience may enhance confidence, perspectives on expanded prescribing remain diverse [22]. Many pharmacists continue to express reservations, particularly regarding independent prescribing, highlighting ongoing concerns about training, professional roles, and the evolving scope of pharmacy practice.

Among pharmacists experienced in expanded roles, including supplementary prescribing models in the United Kingdom, consensus is lacking regarding the necessity of independent prescribing as a progression. Some practitioners argue that collaborative models more effectively support interdisciplinary care, whereas others raise concerns that independent prescribing could result in role overlap or disrupt established team dynamics [22]. Nevertheless, evidence suggests that, with appropriate safeguards, training, and communication, pharmacist prescribing can complement existing roles and enhance access to care.

These differing perspectives underscore the necessity for clear role delineation, robust training frameworks, and policies that facilitate interprofessional collaboration. Over time, pharmacists in Nova Scotia appear to have become increasingly familiar with the prescribing flexibilities under subsection 56(1) of the Controlled Drugs and Substances Act, leading to a gradual increase in uptake. This trend aligns with the typical implementation of new healthcare policies, in which initial uncertainty is subsequently replaced by greater integration into routine practice.

Although the observed differences between urban and rural pharmacists reached statistical significance, their absolute magnitude was minimal. This outcome likely results from the large sample size and the low baseline proportion of pharmacist-prescribed opioid claims, conditions under which even modest changes can achieve statistical significance. Therefore, these findings should be interpreted with caution, as statistical significance does not inherently indicate clinical or practical relevance.

These results indicate relative differences in the proportion of opioid claims attributed to pharmacists, rather than absolute prescribing rates. Although these findings may suggest variation in the adoption of expanded prescribing authority, the real-world implications of these differences are unclear. Furthermore, the observed increase in prescribing does not necessarily indicate either improved or problematic care. This increase may reflect enhanced access during the pandemic; however, it cannot be assessed for appropriateness, dosage, or patient risk.

These findings indicate that a cautious and context-specific approach to expanded pharmacist prescribing is warranted from a policy standpoint. Continuous monitoring, integration of clinical safeguards, and use of more granular data are essential to ensure safe implementation. Additional research that includes absolute prescribing measures and patient-level outcomes is required to clarify the clinical and public health implications of these trends.

Several limitations should be considered when interpreting these findings. First, the ecological and descriptive study design precludes causal inference; therefore, re-sults should be interpreted as population-level associations rather than direct effects of the Controlled Drugs and Substances Act exemption. Second, the analysis did not ac-count for concurrent factors such as pandemic waves, disruptions in healthcare access, or changes in care delivery, all of which may have influenced opioid prescribing patterns during the study period. Third, there is potential for confounding bias because important patient-, prescriber-, and system-level factors were not measured or adjust-ed for in the analysis. These unmeasured variables may have influenced the observed prescribing patterns. Fourth, the absence of a detailed clinical context is a limitation. The data set did not include information on morphine milligram equivalents (MME), prescription duration, or patient-level risk factors, which are important for assessing the intensity, appropriateness, and safety of opioid prescribing. Finally, the outcome was defined as the proportion of total opioid claims, which may be influenced by changes in the denominator rather than true changes in prescribing volume. This in-troduces potential measurement bias and limits the ability to interpret findings as ab-solute increases or decreases in opioid prescribing.

## 5. Conclusions

Pharmacists in Nova Scotia utilized the temporary exemption under subsection 56(1) of the Controlled Drugs and Substances Act during the COVID-19 pandemic to ensure continued access to opioid therapies. The results indicate significant differences in prescribing patterns between urban and rural populations, demonstrating that geographic context influenced the application of these prescribing authorities. Between 19 March 2020 and 30 April 2022, opioid prescribing increased consistently in both groups, reflecting a broader shift in utilization during the pandemic. This upward trend likely resulted from multiple factors, including disruptions to standard care pathways, expanded pharmacist scope of practice, and changing patient needs amid public health restrictions. These findings emphasize the need to account for place-based differences when assessing policy changes and highlight the critical role of pharmacists in sustaining continuity of care during periods of healthcare system strain.

## Figures and Tables

**Table 1:** Estimated policy effect of fitting the time series regression model to the urban and rural claims data from the Nova Scotia Prescription Monitoring Program.

	Urban Claims	Rural Claims
Estimated **mean** claims per week	0.667%	0.201%
95% Confidence Interval of **mean** claims per week	(0.520%, 0.838%)	(0.140%, 0.291%)
Estimated **slope** of claims per week	1.007	1.009
95% Confidence Interval of **slope** of claims per week	(1.003, 1.010)	(1.003, 1.014)

## Data Availability

The datasets presented in this article are not readily available because of confidentiality. Requests to access the datasets should be directed to the corresponding author.

## Abbreviations

The following abbreviations are used in this manuscript:

AIC	Akaike Information Criterion
ARIMA	Autoregressive Integrated Moving Average
CDSA	Controlled Drugs and Substances Act
NSPMP	Nova Scotia Prescription Monitoring Program
SD	Standard Deviation
